# Ischemic Heart Disease: New Insights from Imaging Diagnostic Techniques

**DOI:** 10.1155/2018/5723502

**Published:** 2018-12-23

**Authors:** Andrea Igoren Guaricci, Maurizio Galderisi, Giovanni Aquaro, Nazario Carrabba, Pontone Gianluca

**Affiliations:** ^1^Cardiology Institute, Department of Emergency and Organ Transplantation, University Hospital Policlinico Consorziale of Bari, Bari, Italy; ^2^Department of Advanced Biomedical Sciences, Federico II University, Naples, Italy; ^3^Fondazione G. Monasterio CNR Regione, Pisa, Italy; ^4^Division of Cardiology, Careggi Hospital, Florence, Italy; ^5^Centro Cardiologico Monzino, IRCCS, Milan, Italy

Coronary artery disease (CAD) is the first cause of mortality worldwide and the majority of individuals older than 60 years of age suffer from its consequences. It is ascertained that the epidemiology and social impact of ischemic heart disease (IHD) are huge and big efforts are mandatory in diagnostic and prognostic fields.

Modern and thrilling technology such as coronary computed tomography angiography (CCTA) and cardiac magnetic resonance (CMR) has been emerging and offering the possibility to characterize both the coronary arteries and the myocardium at a very high level of detail.

Our special issue, which had opened for 6 months in the first half of 2018, mainly focused on scientific evidences of advance in cardiac and coronary CT and CMR and included four narrative reviews and two original articles.

Carrabba et al. critically analyzed the National Institute for Health and Care Excellence (NICE) guidelines on chest pain published in 2016 and underscored the key changes provided in comparison to the 2010 version [1]. At first, the previously proposed pretest probability risk score was no longer recommended. Moreover, the guidelines changed approach for patients with low pretest probability and calcium score of zero who should not be considered by default “free from CAD”. Importantly, the new guidelines recommended CCTA as a first-line investigation in all patients with new onset chest pain. The authors argued that few clinical and economic data existed in support of the use of CCTA over all other noninvasive imaging testing especially in patients at intermediate-to-high risk of CAD. Other issues in contrast with the new NICE guidelines recommendation consisted of the limited availability of latest generation scanners in European countries and the negative effects of high cumulative radiation dose exposure from multiple serial CCTA investigations.

Ball et al. described the fractional flow reserve (FFR) derived from CCTA datasets (FFR_CT_) as major advance in cardiovascular imaging, able to provide critical information of coronary tree without exposing the patient to added risk. According to authors' report, invasive FFR measurements represent a guide to percutaneous coronary revascularization and have demonstrated to reduce contrast use, cost, or care and improve outcomes [2]. As being a noninvasive method, FFR_CT_ values are obtained using resting 3D CCTA images through computational fluid dynamics. Several multicenter clinical trials demonstrated the diagnostic superiority of FFR_CT_ over traditional CCTA for the diagnosis of functionally significant CAD and put it in competition with refined diagnostic tools as stress CMR and coronary CT perfusion [3]. Thanks to the high diagnostic accuracy, FFR_CT_ offers the possibility to distinguish between patients who can safely avoid invasive coronary angiography and those patients who require revascularization.

Seitun et al. focused their paper on the new generation multidetector row (≥64 slices) CT systems which allow an accurate assessment of both coronary epicardial stenosis and myocardial CT perfusion imaging at rest and during pharmacologic stress as “one-stop-shop” method [4]. Indeed, this application leads to the comprehensive assessment of both anatomical coronary details and its physiological consequences and represents a valid alternative to FF_CT_. The authors detailed the technical aspects of coronary CT perfusion and, at the same time, pinpointed its strength and limitation points and summarized the evidences about its clinical applications. Indeed, the recent literature suggests that this method is safe and powerful and able to improve the accuracy and the positive predictive value of CCTA alone adding functional information [5]. In their conclusions, Seitun et al. invited to perform large prognostic studies in order to assess if this combined approach might have substantial impact on patients management and costs.

Carità et al. considered in their review that primary prevention of major cardiac events needed a strong implementation for ethic and economic reasons. The authors took into consideration the prognostic value of CCTA in the first half of the manuscript and concluded with the description of the recent literature upon the therapeutic perspective. As well described by the authors, CCTA offers the possibility to study the coronary arteries beyond the assessment of coronary stenosis, evaluating the plaque composition and the presence of positive remodeling. This ability has opened a new scenario about the possibility of estimating the prognostic profile of the single patient [6]. Indeed, together with coronary stenosis severity, which represents a powerful predictor of prognosis in CAD, other elements identified by CCTA have been added as prognostic predictors, such as the spotty calcifications, the low attenuation plaque (<30 HU), and the high positive remodeling index [7]. Therefore, early identification of CAD, characterization of atherosclerotic progression, and assessment of “vulnerable plaque”, sometimes in the context of “vulnerable patient”, are considered by the authors as mandatory endpoints [8]. Afterwards, Carità et al. underscored that research in this field may also advance towards the definition of individualized medical therapy on the basis that statins may delay plaque progression and change some plaque features [9].

Catalano et al. sought to evaluate the added prognostic value of CMR as compared to conventional risk profiling, including clinical history, atherosclerosis risk factors, electrocardiography, and echocardiography, in 465 patients affected by stable CAD who underwent a comprehensive CMR evaluation which consisted of left ventricle dimensions and functioning, late gadolinium enhancement (LGE) and stress perfusion sequences. The authors concluded that LGE and stress perfusion assessment independently predicted MACE beyond conventional risk stratification in this subset of patients. Moreover, authors discussed some critical insight of CMR partially explored from existing literature, such as the prognostic value of LGE in the middle-long term [10] and the importance of a comprehensive CMR assessment, including the stress perfusion acquisition, which may be a useful facility to predict morbidity as well as mortality [11].

Lin et al. aimed to explore the role of the CHADS2 score in the evaluation of carotid atherosclerosis in 109 patients with nonvalvular atrial fibrillation undergoing carotid artery ultrasonography and velocity vector imaging (a parameter reflecting the long-axis longitudinal motion function of carotid arteries) [12, 13]. Interestingly, the authors revealed that carotid arterial structure and function were significantly altered, including increased arterial wall stiffness, decreased elasticity, and aggravated atherosclerosis, in patients with atrial fibrillation and that the burden of carotid atherosclerosis depended on the duration of atrial fibrillation. Finally, they postulated that the CHADS2 score might be useful as a predictor of the extent of carotid atherosclerosis in this subset of patients.
